# ClimCKmap, a spatially, temporally and climatically explicit distribution database for the Italian fauna

**DOI:** 10.1038/s41597-019-0203-6

**Published:** 2019-10-08

**Authors:** Silvio Marta, Michele Brunetti, Gentile Francesco Ficetola, Fabio Stoch, Giovanni Amori, Donatella Cesaroni, Valerio Sbordoni, Antonello Provenzale

**Affiliations:** 1 Department of Environmental Science and Policy, University of Milan, Via G. Celoria 26, 20133 Milan, Italy; 2 Institute of Ecosystem Studies, ISE-CNR c/o Department of Biology, University of Rome ‘Tor Vergata’, Via della Ricerca Scientifica, 00133 Rome, Italy; 3 Institute of Atmospheric Sciences and Climate, ISAC-CNR, 40129 Bologna, Italy; 4 Evolutionary Biology & Ecology - C.P. 160/12, Université Libre de Bruxelles, Avenue F.D. Roosevelt 50, B-1050 Brussels, Belgium; 5 Institute of Research on Terrestrial Ecosystems, IRET-CNR, Viale dell’Università 32, 00185 Rome, Italy; 6 Department of Biology, University of Rome ‘Tor Vergata’, Via della Ricerca Scientifica, 00133 Rome, Italy; 7 Institute of Geosciences and Earth Resources, IGG-CNR, National Research Council, 56124 Pisa, Italy

**Keywords:** Biogeography, Biodiversity

## Abstract

Understanding and counteracting biodiversity losses requires quantitative knowledge on species distribution and abundance across space and time, as well as integrated and interoperable information on climate conditions and climatic changes. In this paper we developed a new biodiversity-climate database for Italy, ClimCKmap, based on the critical analysis, quality estimation and subsequent integration of the CKmap database with several high-resolution climate datasets. The original database was quality-checked for errors in toponym, species name and dating; the retained records were georeferenced and their distribution polygonised via Voronoi tessellation. We then integrated the species distribution information with several high-resolution climatic datasets: average monthly minimum and maximum temperature and total monthly precipitation were reconstructed for each Voronoi cell and year. The resulting database contains 268,977 occurrence records from 8,445 binomials and 16,332 localities, dating between 1680 and 2006 CE. This dataset, fully available at 10.6084/m9.figshare.7906739.v4 and http://hdl.handle.net/21.11125/a91f85cb-befd-4e14-8e83-24f17c4a0491, represents the largest, fully quality-checked, spatially, temporally and climatically explicit distribution database ever assembled for the Italian fauna, now ready for scientific exploitation.

## Abstract (summary)

**Table:** 

Measurement(s)	biodiversity • climate
Technology Type(s)	digital curation
Factor Type(s)	species • geographic location
Sample Characteristic - Environment	key biodiversity area
Sample Characteristic - Location	Italy

Machine-accessible metadata file describing the reported data: 10.6084/m9.figshare.9822593

## Background & Summary

Biological diversity, along with the associated ecosystem services and genetic resources, is essential to support the wealth of human society. In the last decades, international efforts have been made to collect, standardise and share a huge amount of distribution data, e.g. via the Global Biodiversity Information Facility (GBIF; https://www.gbif.org). Despite the efforts to collect as much information as possible and compile comprehensive databases, several limitations are still present^1^ and biodiversity inventories suffer from various types of bias^2^. Sources of bias include the unequal sampling effort due to site accessibility and attractiveness^3,4^, the introduction of data from non-systematic (i.e. opportunistic or occasional) surveys^5^, the inconsistency of sampling methodologies across space and time^6^ and the taxonomical attractiveness (i.e. species or groups receiving particular attention for being rare, beautiful or somehow charismatic)^7^. Additionally, distribution data from biodiversity inventories are often affected by errors in spatial coordinates^8^, taxonomic misidentification or changes in nomenclature^9^ or need retrospective georeferencing prior to use^10,11^. Even considering all these potential limitations, biodiversity inventories still remain a powerful tool for a deeper understanding of ecosystem complexity and for tracking long-term biodiversity trajectories. In this frame, historical data (e.g. properly labelled specimens from private collections, museums or herbaria) represent a crucial source of information on past species distribution and phenology^12,13^. For example, comparing present-day information with data spanning the period of accelerated anthropogenic habitat destruction and climate change (e.g. the nineteenth and twentieth centuries) allowed to detect local extinctions and altitudinal shifts following human disturbance or recent climate change^14–16^.

Distribution data from collections frequently exhibit high resolution at both the spatial (collection locality) and temporal (complete date or year) scales; such biodiversity inventories are intrinsically spatially-accurate multi-temporal datasets, spanning over wide areas and temporal intervals. Comparable multi-temporal fine-grained abiotic data are thus required to shed light on the patterns of biodiversity response to environmental changes, and to identify the processes underlying such responses^17^. During the past decades, a substantial set of climatologies has been made available to researchers dealing with drivers of species distribution, but each of the most popular climate datasets clearly has some limitations. WorldClim^18,19^ has very broad spatial extent (global) and high resolution (30 arcsec), but lacks of temporal extent (multi-year climatologies: 1950–2000 and 1970–2000, respectively). On the contrary, datasets such as CRUT4^20^, CRU TS 4.02^21^, GISSTEMP^22^, NOAA^23^ and BEST^24^ are global, providing gridded anomalies across the World at monthly resolution with a high temporal extent (first year ranging from mid-18^th^ century for BEST to 1901 for CRU TS 4.02), but with quite coarse spatial resolution (ranging from 5° to 0.5°). Finally, the recently published CHELSA^25^ combines the high spatial extent and resolution of WorldClim (worldwide at 30 arcsec) with the high temporal resolution of CRU TS (monthly means), but is temporally limited to 1979–2013. A major issue of all these datasets is that they are originated from the broad-scale interpolation of data from weather stations, and can be locally inaccurate. Finally, Deblauwe *et al*.^26^ showed that such global datasets have fluctuating performances across the globe, and proposed the use of remotely sensed data to obtain more accurate climatologies, particularly in areas with a limited number of ground stations. Unfortunately, both the Deblauwe dataset and remotely-sensed data are temporally limited to the last forty years.

Our aim here was to produce and share a climatically explicit database on the distribution of Italian fauna with high spatial and temporal resolution. Based on GBIF data, Italy stands out among western countries for the low completeness of its species inventory^27^, even if it is one of the most biodiverse countries in Europe. Italy is formally neither a voting nor an associated participant of GBIF (https://www.gbif.org/the-gbif-network); consequently, there was no massive data contribution to the GBIF database from Italian institutions to date^28^. To fill this gap, in this work we updated, cleaned and georeferenced with greater precision raw distribution data from the extensive database for the Italian fauna (CKmap 5.3.8; ref.^29^), and integrated it with a high-resolution reconstruction of monthly temperature and precipitation in the location and at the time specimens were collected. The dataset compiled here represents the most spatially and temporally comprehensive and accurate faunal distribution database available for the Italian fauna, as well as one of the first attempts to finely yet massively reconstruct climate conditions under which biodiversity data were collected over more than two centuries.

## Methods

CKmap 5.3.8 (ref.^29^; English edition – version 5.4) reports the taxonomical data and the occurrence in Italy of over 10,000 terrestrial and freshwater animal species^30^. Species distribution was mapped attributing each record (i.e. the occurrence of a species in a single location) to the 10 × 10 km UTM cell (ED50 datum, MGRS system) on the basis of a gazetteer. The gazetteer stored in the database (available for download at http://www.faunaitalia.it/documents/TCI.zip) included 46,961 toponyms taken from the “Touring Club Italiano” (TCI) atlas, accurately georeferenced using topographic maps of Italy at the scale 1:25,000^30^.

The geographic cleaning phase included quality checks for both CKmap and the reference gazetteer. All toponyms were checked for double spacing and/or additional spacing at the beginning and at the end of the string; all the records with an empty locality field were discarded. In the gazetteer, the precision of coordinates was indicated with the letters A, F and G^30^. All G records were excluded, given they represent the centroid of broad spatial polygons (i.e., mountain ranges or rivers). A one-to-many join then allowed linking each of the remaining toponyms (A and F; i.e. individual locations) to the corresponding distribution records. An R code allowing partial matches (i.e. allowing up to 5% of letter deletion, insertion or substitution) was run on non-matching records and putative matches were carefully verified by hand prior to assignment. The distribution of the retained records was polygonised via a Voronoi tessellation using the deldir R package^31^ and the resulting Voronoi diagram was clipped using coastline and administrative boundaries. We used this approach because database compilers described each collection locality using the closer toponym^30^. For each Voronoi cell, spatial precision was calculated as the distance between the sample point (toponym) and the farthest vertex (i.e. the positive pole, for bounded cells). The dimension of each Voronoi cell, and hence the record precision, obviously depends on the local toponym density.

The database cleaning consisted in the deletion of inaccurate records. All the records reporting an inaccurate taxonomical classification (e.g. using open nomenclature^32^ instead of the Linnean binomials) were discarded. The remaining binomials were cleaned by removing subspecific classification and checking for extra spacing and typos using the above-mentioned approach. Furthermore, all records without collection date were discarded and the remaining records were checked for agreement with publication year. All the records with publication year (if any) later than collection year were retained; we also retained all the records without publication year, which represented unpublished collection specimens. We finally removed duplicate records (i.e. occurrence records of the same taxon from the same location and year), except when they were from different sources or were collected at different elevations.

For each retained record, we reconstructed average monthly minimum and maximum temperature and total monthly precipitation exploiting the huge amount of meteorological observations available for Italy since the 18^th^ century^33^. We used the anomaly method for climate reconstruction^34^, which is based on the independent reconstruction of the climatologies (i.e. the climate normal over the standard reference period) and the deviations from them (i.e. the anomalies with respect to the same baseline period). Climatologies are characterized by strong spatial gradients and a large number of weather stations is necessary to capture them, even if available for a short period. Consequently, an interpolation technique that exploits the dependence of climate normals on orography and other geographic parameters is necessary to reconstruct such climatologies^35–37^. Anomalies, on the other hand, are linked to climate change and variability; for this reason, they are usually characterized by higher spatial coherence^34^. A limited number of weather stations can therefore be sufficient to capture spatial patterns through simpler interpolation methods, but it is pivotal to use long-term time series to have a satisfactory temporal coverage over the past. These series must in turn be corrected for errors deriving from the history of the stations (changes of station and instrument location, instrument replacements, changes in observation protocols, etc.) which could interfere with the actual climate signal^33,38,39^. For each record, we reconstructed the climate information of the Voronoi cell centre using average cell elevation following Brunetti *et al*.^40^. For each centre we first estimated the climate normals calculating a weighted linear regression of the data from nearby stations as a function of elevation. We assigned greater weights to the stations with elevation and topographic position similar to that of the location of interest, as derived from a 30 arcsec resolution digital elevation model^36,37^. Anomalies were interpolated at the same locations exploiting an improved version of the dataset presented in Brunetti *et al*.^33^ and then, by combining anomalies and climatologies, temporal series of temperature and precipitation in absolute values were obtained for each record location. Bioclimatic variables were finally calculated on monthly temperature and precipitation using the dismo R package^41^. All the above-mentioned analyses were run using custom R and Fortran codes.

## Data Records

**Fig. 1:**
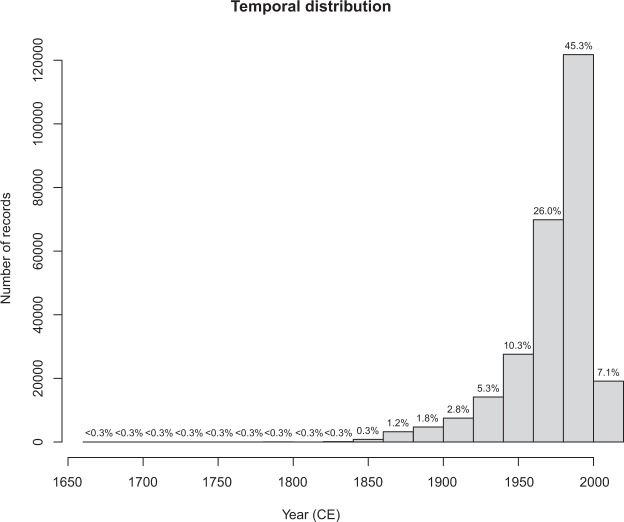
Temporal distribution of the records from ClimCKmap. Total number of records: 268,977 from 8,445 binomials and 16,332 localities. Temporal coverage: 1680–2006 CE. The numerals above each bar indicate the percentage of records falling in that time period.

The final dataset includes 268,977 occurrence records deriving from 8,445 binomials and 16,332 localities, dating between 1680 and 2006 CE (Fig. 1). A complete description of the database structure is reported in the Online-only Table 1.

The full database is made accessible at 10.6084/m9.figshare.7906739.v4 ^42^ and http://hdl.handle.net/21.11125/a91f85cb-befd-4e14-8e83-24f17c4a0491.

## Technical Validation

**Fig. 2:**
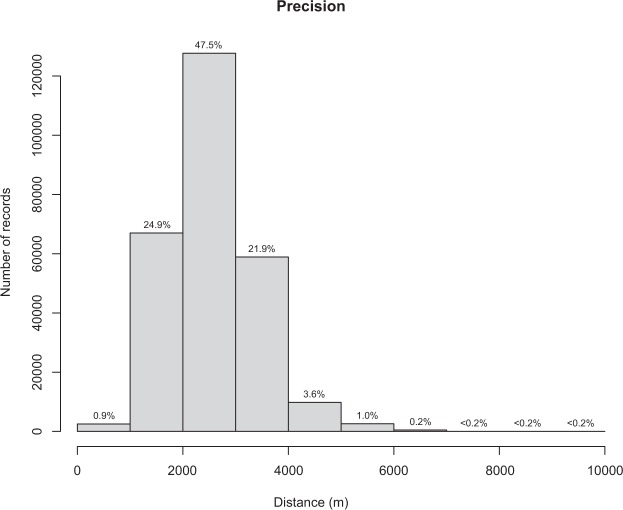
Distance between sample point and the farthest vertex of the Voronoi cell (precision). Median = 2480 m; 95% confidence interval = 1251–4474 m. The numerals above each bar indicate the percentage of records falling in that distance class.

The starting dataset (CKmap 5.3.8; ref.^29^) included 548,868 occurrence records from 10,132 taxa. After taxonomic simplification and cleaning, 544,764 records from 10,064 binomials (i.e. species) were retained. The subsequent geographic cleaning and georeferencing phase allowed assigning coordinates to 470,232 records from 19,574 localities. The removal of non- or dubiously-dated records restricted the size of the resulting dataset to 277,310 records. The removal of duplicates led to a final dataset including 268,977 distribution records deriving from 8,445 binomials and 16,332 localities, dating between 1680 and 2006 CE (Fig. 1). The georeferencing methodology returned satisfactory results in term of precision (Fig. 2; median accuracy = 2480 m; for 95% of records accuracy was between 1251 and 4474 m). Climate reconstructions did not allow assigning climate data to only a small fraction of the total dataset (894 records dating between 1680 and 1921 CE; 0.3%) due to the lack of meteorological data (pre-instrumental period) or the incompleteness of the local time series. Monthly means of minimum and maximum temperature and total precipitation were thus reconstructed for the remaining part of the records (268,083 records dating between 1790 e 2006 CE).

## Usage Notes

In the last two centuries, biodiversity has been facing abrupt climate and environmental changes globally, mainly due to human activities. Huge efforts have been spent to expand our knowledge of species distribution worldwide, but the picture is still far from complete. In fact, apart from rare exceptions^43^, low taxonomic coverage and temporal gaps still limit our understanding of ecosystem functioning under climate change and biodiversity loss. The dataset we compiled and presented here aimed at moving a step forward to fill this gap, by providing spatially, temporally and climatically accurate distribution information for the fauna of one of the most biodiverse countries in Europe. ClimCKmap is currently the largest spatially, temporally and climatically explicit distribution database ever assembled for the Italian fauna. Future research should consider exploiting the database, for example deriving empirical relationships between climate change and biodiversity loss and using them to evaluate impacts of future climate change scenarios, as well as to identify climate-biodiversity change hotspots. Proper use of the data will also allow for developing knowledge-based biodiversity conservation measures.

This database has of course some potential issues that have to be taken into account when working with it. First, we opted for retaining the original classification and taxonomy, i.e. a revised form of the checklist of the Italian fauna^44^. Additionally, some authors reported biases due to spatially unequal sampling effort^45,46^, as well as temporal and taxon-specific biases in the completeness of the database^46^. Researchers interested in exploiting the database should thus evaluate case by case if a taxonomic updating for the group under analysis is needed, and be aware of the potentially (spatially and/or temporally) biased nature of the database itself.

## Data Availability

Custom R and Fortran codes used to run the analyses, together with an example dataset are available at 10.6084/m9.figshare.7906739.v4 (ref.^42^).

## Online-only Table

**Online-only Table 1:** Database structure.

	Field name	Unit	Description
Taxonomy			
	phylum		Original, taxonomic classification
	class		Original, taxonomic classification
	order		Original, taxonomic classification
	family		Original, taxonomic classification
	species		Original, taxonomic classification
Binomial			
	binomial		Simplified binomial (genus and species only)
Administrative			
	reg		Region
	prov		Province
	loc		Collection locality
Source			
	source		Original, collection or publication information
Elevation			
	elev	m a.s.l.	Original, elevation
	srtm	m a.s.l.	Per-cell average elevation (SRTM, resolution: 90 m)
Year			
	year	year	Original, year (CE)
Coordinates			
	x	m	Easting - EPSG:3035 (ETRS89/ETRS-LAEA)
	y	m	Northing - EPSG:3035 (ETRS89/ETRS-LAEA)
	mgrs		Original, UTM ED50 10 × 10 km cells, MGRS format
Precision			
	precision	m	Distance - sample point to farthest vertex
Climate			
	bio01	°C	Mean annual temperature
	bio02	°C	Mean diurnal range (mean of max temp - min temp)
	bio03		Isothermality ((bio02/bio07) * 100)
	bio04	°C	Temperature seasonality (standard deviation * 100)
	bio05	°C	Max temperature of warmest month
	bio06	°C	Min temperature of coldest month
	bio07	°C	Temperature annual range (bio05–bio06)
	bio08	°C	Mean temperature of the wettest quarter
	bio09	°C	Mean temperature of driest quarter
	bio10	°C	Mean temperature of warmest quarter
	bio11	°C	Mean temperature of coldest quarter
	bio12	mm	Annual precipitation
	bio13	mm	Precipitation of wettest month
	bio14	mm	Precipitation of driest month
	bio15		Precipitation seasonality (coefficient of variation)
	bio16	mm	Precipitation of wettest quarter
	bio17	mm	Precipitation of driest quarter
	bio18	mm	Precipitation of warmest quarter
	bio19	mm	Precipitation of coldest quarter

Fields whose description starts with “Original” contain data that were not modified during dataset building. The complete distribution database (ClimCkmap) is composed by 268,977 records from 8,445 binomials and 16,332 localities, dating between 1680 and 2006 CE.
